# Lossless Image Compression Using Context-Dependent Linear Prediction Based on Mean Absolute Error Minimization

**DOI:** 10.3390/e26121115

**Published:** 2024-12-20

**Authors:** Grzegorz Ulacha, Mirosław Łazoryszczak

**Affiliations:** Faculty of Computer Science and Information Technology, West Pomeranian University of Technology in Szczecin, ul. Żołnierska 49, 71-210 Szczecin, Poland

**Keywords:** entropy coding, iterative reweighted least squares, context-dependent coding, deep learning, lossless image coding

## Abstract

This paper presents a method for lossless compression of images with fast decoding time and the option to select encoder parameters for individual image characteristics to increase compression efficiency. The data modeling stage was based on linear and nonlinear prediction, which was complemented by a simple block for removing the context-dependent constant component. The prediction was based on the Iterative Reweighted Least Squares (*IRLS*) method which allowed the minimization of mean absolute error. Two-stage compression was used to encode prediction errors: an adaptive Golomb and a binary arithmetic coding. High compression efficiency was achieved by using an author’s context-switching algorithm, which allows several prediction models tailored to the individual characteristics of each image area. In addition, an analysis of the impact of individual encoder parameters on efficiency and encoding time was conducted, and the efficiency of the proposed solution was shown against competing solutions, showing a 9.1% improvement in the bit average of files for the entire test base compared to *JPEG-LS*.

## 1. Introduction

In many image processing and archiving applications, the storage of lossless data is required. Significant applications of lossless compression of images and video sequences include archiving of 2D, 3D, and 4D medical images (3D video sequences) [1,2,3,4,5], astronomical images, and compression of satellite images [6,7]. In addition, lossless mode is often required in the graphic processing of photos and advertising materials and the post-production of television broadcasts and films [8].

Modern compression methods usually take into account two steps: data decorrelation and compression by one of the efficient entropy methods, among which the most effective are arithmetic coding, Huffman coding, and its specific variations (Golomb codes, Rice codes) [9]. The decorrelation is designed to significantly reduce data redundancy resulting from the high level of correlation between neighboring pixels. For this purpose, simple prediction methods (*JPEG-LS* [10], *CALIC* [11]), both linear and non-linear, described in works [12,13,14,15,16], are most often used. There are also other approaches, such as wavelet transforms (*JPEG 2000* [17] and *SPIHT* [18]), where their specific features are used to record image data efficiently.

The highest efficiency of lossless compression is those with high implementation complexity, which belongs to the class of time-symmetric (where the decoding time is as long as the encoding time). In such cases, the methods are based on linear prediction models with backward adaptation, using mechanisms known from the literature such as *RLS* [19], *OLS* [20,21,22], or *WLS* [23,24], where also the coding of each successive pixel is accompanied by a procedure for adapting or redetermining the coefficients of the linear predictor. A cascaded combination of predictors (with backward adaptation) of the *WLS*/*NLMS* type achieves the highest compression efficiency, as shown in [23], but this is associated with decoding times that are too long. In addition, a branch of lossless image compression methods based on deep learning using neural networks has been developed recently. So, we have devoted a separate Section 4.2 to this issue, pointing out some disadvantages of this solution.

In this work, the authors proposed lossless compression of images with asymmetric encoding and decoding times with relatively short decoding times (since the encoding operation is usually performed once and the decoding multiple times) while maintaining high compression efficiency. This is made possible using linear prediction with forward adaptation (Section 2.1) supported by fast, nonlinear prediction based on simplified blending of constant predictors (Section 2.2). Section 2.3 briefly characterizes the entire data processing in the encoder and decoder. At the same time, the rest of the paper focuses on the main block using linear prediction (an improved *IRLS* algorithm to minimize mean absolute error described in Section 2.4) and how to increase prediction efficiency by introducing context switching (Section 3.1). Section 3.2 analyzes the impact of the proposed method’s parameter selection on its efficiency and encoding and decoding times. Section 4 compares the compression efficiency with other codecs known from the literature.

## 2. Using Linear Prediction for Image Compression

### 2.1. Basics of Predictive Modeling

In the case of two-dimensional signals such as images, the data modeling stage involves removing as much of the causal information occurring between adjacent pixels as possible. Therefore, the predicted value ${\hat{x}}_{n}$ ([] denotes rounding operation in Formula (1)) of the encoded pixel $x_{n}$ is determined, and then the differences between them are encoded:

(1) $$e_{n}=x_{n}-\left[{\hat{x}}_{n}\right].$$

**Figure 1:**
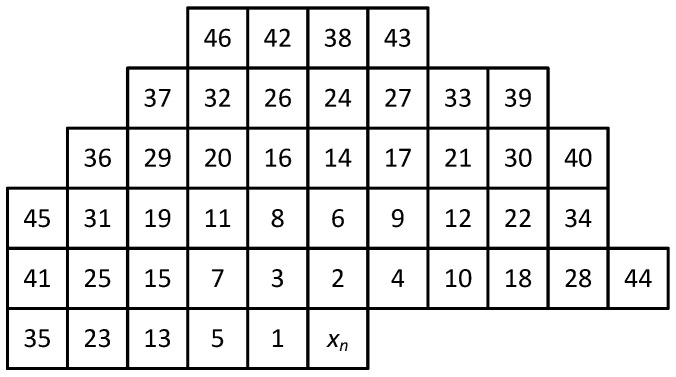
Numbering of neighborhood pixels relative to the currently encoded pixel $x_{n}$.

Such differences, $e_{n}$, referred to as prediction errors, are often small values oscillating near zero with a probability distribution close to the Laplace distribution. A linear predictor of order *r* can be used as the predicted value:

(2) $${\hat{x}}_{n}=\sum_{j=1}^{r}b_{j}\;\cdot \;P\left(j\right),$$

where the elements $b_{j}$ are the prediction coefficients that make up the vector $B=\left[b_{1},b_{2},\ldots ,b_{r}\right]$, while P(j) denotes the *j*th pixel adjacent to the currently encoded $x_{n}$ (see Figure 1 [23] showing the 46 numbered nearest neighbors of the encoded pixel P(j), where a given *j*th number indicates a pixel with a value of P(j) and a prediction error of e(j)). This is a relative numbering referring to the currently encoded pixel (with an index of 0; hence, you can use the relative assignment of $x_{n}=P\left(0\right)$ and $e_{n}=e\left(0\right)$ for the current prediction error). The image is encoded row by row, from left to right; hence, if we consider the *n*th time moment, the currently encoded pixel $x_{n}=P\left(0\right)$, and the pixel encoded at a time (n−2), i.e., $x_{n-2}$, has a relative index P(5), according to Figure 1.

The simplest predictors are just the values of individual pixels (with r=1 and $b_{1}=1$), e.g., ${\hat{x}}_{n}=P\left(4\right)$ means that the predicted value of the encoded pixel $x_{n}$ is the value of the upper right neighbor.

### 2.2. Non-Linear Predictive Model Support

The solution proposed here assumes an asymmetric approach between encoding and decoding times. Thus, increasing the implementation complexity of the encoder becomes acceptable by introducing forward adaptation, which requires access to the data of the whole image for multiple preliminary data modeling with parameter tuning before the final encoding stage. On the other hand, the decoding process is expected to have a reasonably short decoding time. This calls for a predictive method with low implementation complexity. The compromise solution is to use linear prediction enhanced by two additional techniques to obtain an auxiliary predicted value based on nonlinear prediction. This applies to *GAP*_+_ (Gradient-Adjusted Predictor) [25] and *GBSW*_+_ (Gradient-Based Selection and Weighting) [26], which, despite using backward adaptation, have low implementation complexity. The “+” symbol in the name denotes enhancements to the original algorithms, which we discussed in [27]. The final form of the predicted value, which is an expansion of the Formula (2) for the encoder proposed in this work, is determined as follows:

(3) $${\hat{x}}_{n}=b_{1}\;\cdot \;{\hat{x}}_{GBSW_{+}}+b_{2}\;\cdot \;{\hat{x}}_{GAP_{+}}+\sum_{j=3}^{r}b_{j}\;\cdot \;P\left(j-2\right).$$

This means that, for example, with order r=14, in addition to the auxiliary predicted values ${\hat{x}}_{GBSW_{+}}$ and ${\hat{x}}_{GAP_{+}}$, we also use the 12 nearest neighbors whose Euclidean distance from the encoded pixel $x_{n}$ is less than 3 (see Figure 1).

After an in-depth analysis of the *GBSW* method [26], its strong affinity with the simple blending version of the [28,29] predictors becomes apparent, as will be demonstrated below. This leads to the possibility of further improving *GBSW*_+_ relative to the version presented in the work of [27], where a weighted average of two of the four nearest neighborhood pixels constituting the set of potential sub-predictors is determined as the predicted value of ${\hat{x}}_{GBSW_{+}}$. The predicted value depends on the initial determination of the two currently most minor of the four gradients $d_{W}$, $d_{N}$, $d_{NW}$ and $d_{NE}$ (associated, respectively, with simple predictors of order one: P(1), P(2), P(3) and P(4) referred to hereafter as sub-predictors), where the subscripts indicate the direction of the gradient: *W*—west, *N*—north, NW—northwest and NE—northeast.

For example, if the two smallest values are $d_{W}$ and $d_{N}$, the prediction value is determined as follows:

(4) $${\hat{x}}_{GBSW_{+}}=\frac{d_{W}\;\cdot \;P\left(2\right)+d_{N}\;\cdot \;P\left(1\right)}{d_{W}+d_{N}}.$$

These gradients are a weighted average of the absolute differences between adjacent nearest-neighbor pixels in a specific direction (to the nearest $45^{{}^{\circ}}$). These differences can also be viewed as prediction errors of the individual four sub-predictors from the set of P(1),P(2),P(3),P(4) applied to the previously coded pixels. For example, if at the *n*th time instant the component of the $d_{NE}$ gradient is $\left|P(5)-P(3)\right|$, this corresponds to the prediction error $\left|e{\left(5\right)}_{NE}\right|$ (where $e{\left(j\right)}_{NE}$ specifies the *j*th prediction error using the northeast-type sub-predictor at previous time instants). It means that according to Figure 1, the northeast type predictor for pixel P(5) at time (n−2) was its upper right neighbor ${\hat{x}}_{n-2}=P\left(3\right)$, then the prediction error is $e{\left(5\right)}_{NE}=P\left(5\right)-P\left(3\right)$.

In such notation, the gradients (multiplied by 30 to dispense with floating-point operations) take the following form:

(5) $$\begin{array}{cc} d_{W}= & 3\;\cdot \;(2\;\cdot \;(\left|e{\left(1\right)}_{W}\right|+\left|e{\left(2\right)}_{W}\right|+\left|e{\left(3\right)}_{W}\right|+\\ \; & \left|e{\left(4\right)}_{W}\right|)+\left|e{\left(6\right)}_{W}\right|+\left|e{\left(9\right)}_{W}\right|)\\ d_{N}= & 3\;\cdot \;(2\;\cdot \;(\left|e{\left(1\right)}_{N}\right|+\left|e{\left(2\right)}_{N}\right|+\left|e{\left(3\right)}_{N}\right|+\\ \; & \left|e{\left(4\right)}_{N}\right|)+\left|e{\left(5\right)}_{N}\right|+\left|e{\left(7\right)}_{N}\right|)\\ d_{NW}= & 5\;\cdot \;(2\;\cdot \;\left(\left|e{\left(1\right)}_{NW}\right|+\left|e{\left(2\right)}_{NW}\right|\right)+\\ \; & \left|e{\left(3\right)}_{NW}\right|+\left|e{\left(4\right)}_{NW}\right|)\\ d_{NE}= & 5\;\cdot \;(2\;\cdot \;\left(\left|e{\left(2\right)}_{NE}\right|+\left|e{\left(5\right)}_{NE}\right|\right)+\\ \; & \left|e{\left(1\right)}_{NE}\right|+\left|e{\left(3\right)}_{NE}\right|) \end{array}.$$

The same principle should be adopted to compare the predictive blending method with $GBSW_{+}$: only two of the four sub-predictors are of interest (in the basic approach, the blending method uses all available sub-predictors from the defined set). Then (assuming that the *n*th time instant most minor gradients are, for example, $d_{W}$ and $d_{N}$) for sub-predictors P(1) and P(2), we obtain the following formula:

(6) $${\hat{x}}_{n}=\frac{\frac{1}{d_{W}^{\alpha }}\;\cdot \;P\left(1\right)+\frac{1}{d_{N}^{\alpha }}\;\cdot \;P\left(2\right)}{\frac{1}{d_{W}^{\alpha }}+\frac{1}{d_{N}^{\alpha }}}.$$

Here, we use the principle that the predicted value in the method of predictor blending is their weighted average with weights inversely proportional to the average level of prediction errors. The corresponding values are $d_{W}$ and $d_{N}$. In addition, the sum of the weights must be equal to 1.

Simplifying, at α=1, the Formula (6) reduces to the form of the Formula (4) known from the *GBSW*_+_ method.

With the introduction of the additional parameter α, after performing experiments using the final Formula (3), more favorable results were obtained in this work with α=2, which led to the modification of Formula (4) to the form:

(7) $${\hat{x}}_{GBSW_{+}}=\frac{d_{W}^{2}\;\cdot \;P\left(2\right)+d_{N}^{2}\;\cdot \;P\left(1\right)}{d_{W}^{2}+d_{N}^{2}}.$$

When the denominator in Formula (7) is equal to 0, then the predicted value is obtained from the *GAP*_+_ model.

### 2.3. Component Blocks of the Proposed Codec

**Figure 2:**
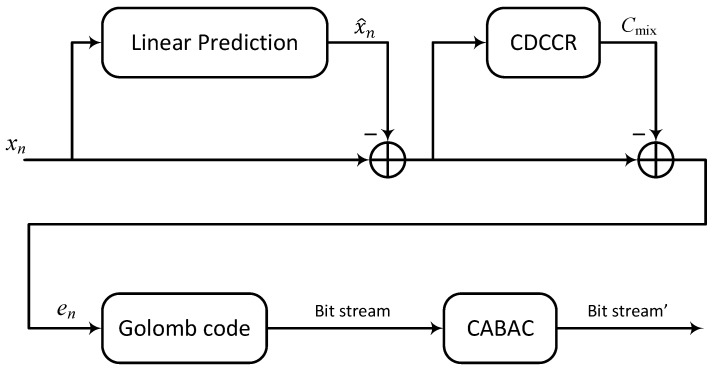
Block diagram of the cascade coding proposed in this paper.

The solution proposed in this work was based on a cascade approach (see Figure 2), in which, in addition to the predicted value calculated using linear prediction, an additional *CDCCR* (Context-Dependent Constant Component Removing) block was used to remove the constant component $C_{mix}$ associated with a specific context (detailed description of *CDCCR* in [30]). This idea has been used before in codecs such as *JPEG-LS* and *CALIC*. Removing the constant component $C_{mix}$ associated with one of the 1024 contexts results in a slight modification of Formula (1) to the form:

(8) $$e_{n}=x_{n}-\left[{\hat{x}}_{n}+C_{mix}\right],$$

where ${\hat{x}}_{n}$ is calculated from the Formula (3).

The final blocks of cascade processing are used to efficiently encode prediction errors using an adaptive Golomb encoder and a binary arithmetic encoder (details are discussed in the paper [23]). Algorithms 1 and 2 below show the encoder and decoder processing, respectively.
**Algorithm 1** Encoder data processing1:For each successively encoded pixel $x_{n}$:2:Calculate the predicted value (Formula (3)—stage 1) and prediction error $e_{n}$ after considering context-dependent constant component $C_{mix}$ (Formula (8)—stage 2).3:Convert the prediction error value $e_{n}$ to the bit sequence using adaptive Golomb coder (stage 3).4:Encode the bit sequence from stage 3 using adaptive binary arithmetic coder.5:Return to step 2 if there are still pixels to encode.

**Algorithm 2** Decoder data processing
1:For each successively decoded pixel $x_{n}$:2:Decode the input bit sequence using adaptive binary arithmetic coder and obtain a word of the Golomb code.3:Convert the Golomb word to the prediction error value $e_{n}$.4:Calculate the predicted value according to (3) and the $C_{mix}$ value, and add these values to $e_{n}$ obtaining decoded pixel value $x_{n}$.5:Return to step 2 if there are still pixels to decode.


In this work, we will mainly focus on the detailed aspects of the first block of the encoder related to linear prediction, discussing the method of fast mean absolute error minimization in Section 2.4.1 and the use of context-dependent linear prediction in Section 3. The proposed solution uses forward-adaptive prediction, where at the stage of calculating prediction coefficients, we have access to the entire encoded image. This allows us to achieve a relatively fast decoding process.

### 2.4. Fast Minimization of Mean Absolute Error

Although it has been widely accepted that for images, determining prediction coefficients using the Minimum Mean Square Error (MMSE) method yields satisfactory results [9,23], in the paper [27], we have shown that an even smaller bit average can be obtained using other methods, including after using mean absolute error minimization. We analyzed a brute-force method with exponential complexity against the number of repetitions of precoding, a method based on selective iterative search with polynomial complexity, and a method with a fixed number of five repetitions of precoding based on a modified form of *ALCM*_+_ (Activity Level Classification Model). The latter method makes it possible to reduce the bit average’s value relative to the classic MMSE approach, but it is pretty cumbersome. This is because for the time of adaptive calculation of prediction coefficients, an internal transformation of data from the model of order *r* to a higher order $r_{ALCM}$ is required, and in the final stage, it is necessary to use an inverse transformation (individually, designed for the pair $r,r_{ALCM}$). Two examples for $r=14,\;r_{ALCM}=27$ and $r=24,\;r_{ALCM}=43$ are presented in the paper [27]. The second disadvantage of this solution is that it requires more precision for saving the prediction coefficients (which must be passed to the decoder in the file header) than using classic MMSE. Assuming that the prediction coefficient is in the range [−1.999,1.999], we can store each of the coefficients on some small number of (N+2) bits (allocating one bit each for saving the sign of the number and the integer value, respectively, and *N* bits of precision for saving the fractional part of the coefficient).

The modified *IRLS*_+_ method is devoid of these disadvantages, allowing the calculation of a predictive model of any order in less time, and saving the prediction coefficients requires a precision of only N=9 bits of the fractional part, which is 3 bits less per coefficient than in the *ALCM*_+_-based solution.

#### 2.4.1. Iterative Reweighted Least Squares Algorithm

Reference [31] proposed a minimum mean absolute error (*MMAE*) to obtain better results (when the image was divided into 8×8 squares) compared to using *MMSE*. To determine the predictive model, we do not need to know the optimal solution that guarantees the minimization of mean absolute error, and one of the approximate solutions will suffice. For this purpose, instead of using improved simplex algorithms, a method can be used that uses the classical *MMSE* by reducing it to a Weighted Least Squares (*WLS*) minimization problem in an iterative manner, allowing a suboptimal solution minimizing the mean absolute error to be obtained relatively quickly. To be more specific, it is *IRLS* with the parameter p=1.

*IRLS* allows for an approximate solution that minimizes the average error in the *Q* data area according to the $l_{p}$ norm [32]:

(9) $${∥e∥}_{p}={\left(\sum_{n\in Q}{\left|e_{n}\right|}^{p}\right)}^{1/p},$$

by reducing the issue to solving a WLS problem that offers relatively low computational complexity:

(10) $${∥e∥}_{p}={\left(\sum_{n\in Q}{\left|e_{n}\right|}^{\left(p-2\right)}\;\cdot \;{\left|e_{n}\right|}^{2}\right)}^{1/p}.$$

We can perform an approximate minimization for any $l_{p}$ norm using an iterative expression minimization algorithm:

(11) $${∥e∥}_{p}={\left(\sum_{n\in Q}w_{n}^{2}\;\cdot \;{\left|e_{n}\right|}^{2}\right)}^{1/p}.$$

For this purpose, we set the weights $w_{n}=1$ in the first iteration and minimize the weighted mean squared error following the Formula (11). We obtain this by solving the matrix equation (using Cholesky decomposition):

(12) $$B=R^{-1}\;\cdot \;P,$$

where R is a square matrix of r×r elements R(j,i) such that:

(13) $$R\left(j,i\right)=\sum_{n\in Q}w_{n}^{2}\;\cdot \;y_{n,i}\;\cdot \;y_{n,j},$$

at j={1,2,…,r}, i={1,2,…,r} while P is a vector of r×1 elements P(j) such that:

(14) $$P\left(j\right)=\sum_{n\in Q}w_{n}^{2}\;\cdot \;x_{n}\;\cdot \;y_{n,j},$$

where $x_{n}$ specifies the value of the next *n*th encoded pixel.

In the proposed solution, using an extended prediction model according to Formula (3), we use two nonlinear predicted values ${\hat{x}}_{GBSW_{+}\left(n\right)}$, ${\hat{x}}_{GAP_{+}\left(n\right)}$ and (r−2) nearest neighbor pixels (see Figure 1); therefore, the vector $Y_{n}=\left[y_{n,1},y_{n,2},\ldots ,y_{n,r}\right]$ can be represented as

$\left[{\hat{x}}_{GBSW_{+}\left(n\right)},{\hat{x}}_{GAP_{+}\left(n\right)},P_{n}\left(1\right),P_{n}\left(2\right),\ldots ,P_{n}\left(r-2\right)\right]$.

In each subsequent iteration of the *IRLS*, the weights are determined using the $e_{n}^{old}$ errors obtained from using the linear prediction model obtained from the previous iteration, using the formula:

(15) $$w_{n}={\left|e_{n}^{old}\right|}^{(p-2)/2}.$$

Each successive iteration in the *IRLS* algorithm brings us closer to the expected minimization accuracy, with minimization of the final bit average $L_{avg}$ obtained after taking into account the header data (the cost of saving the prediction coefficients) and entropy coding of prediction errors playing a key role. For eight-bit image data at p=1, good results are obtained after only 8–10 iterations. In the case of p=2, we obtain the classic minimization of the mean-square error, in which case only the first iteration is sufficient.

To minimize the mean absolute error in *IRLS*, we set the parameter p=1. Then, substituting the Formula (15) into (11), we obtain the classical *WLS* problem, with which the approximate minimization of the mean absolute error can be obtained:

(16) $${∥e∥}_{1}=\sum_{n\in Q}\frac{1}{\left|e_{n}^{old}\right|}\;\cdot \;{\left|e_{n}\right|}^{2}\approx \sum_{n\in Q}\left|e_{n}\right|.$$

Particular attention should be paid to prediction errors oscillating near zero, and protection against dividing by 0 should be made. With eight-bit data, where the absolute error is in the range $\left|e_{n}^{old}\right|\in \left[0,255\right]$, it works well to introduce a limiter $\left|e_{n}^{old}\right|\leftarrow \max\left\{0.6,\left|e_{n}^{old}\right|\right\}$ protecting against excessive weights for errors close to zero ($\left|e_{n}^{old}\right|<0.6$). The value of 0.6 was chosen experimentally (with an accuracy of 0.1) to minimize the bit average of the set of learning images. Finally, the approximate solution to minimize the average error in the *Q* data area according to the $l_{1}$ norm takes the form:

(17) $${∥e∥}_{1}=\sum_{n\in Q}\frac{1}{\max\left\{0.6,\left|e_{n}^{old}\right|\right\}}\;\cdot \;{\left|e_{n}\right|}^{2}.$$

It should be noted that at the stage of determining successive iterations, the prediction values (Formula (3)) are not rounded to an integer when using the Formula (1), which increases the computational convergence. Only after the final form of the predictor B has been determined does the differential image coding procedure use Formula (8) to encode the total prediction errors.

#### 2.4.2. Improvements to the *IRLS* Algorithm

Our contribution to accelerating the convergence of *IRLS* (in terms of the approximate solution and eight-bit data) in the case of mean absolute error minimization (p=1) is to replace the weight $w_{n}^{2}$ (Formula (15)) inversely proportional to the absolute value of the prediction error by an exponential function $0.8^{e_{n}^{old}}$. Formula (17) is changed to the form:

(18) $${∥e∥}_{1}=\sum_{n\in Q}0.8^{e_{n}^{old}}\;\cdot \;{\left|e_{n}\right|}^{2}.$$

This halved the number of iterations in the *IRLS* to just five and eliminated problems of dividing by 0. The value of 0.8 was selected by experiment (with an accuracy of 0.01) to minimize the bit average of the set of learning images.

If we assume that the weights in Formulas (17) and (18) are treated as some kind of probability density functions ($f_{1}\left(x\right)$ and $f_{2}\left(x\right)$, respectively) over the range of values of the variable *x* in the interval [0,255], then we get a distribution:

(19) $$f_{1}\left(x\right)=\frac{1}{a\;\cdot \;\max\left\{0.6,x\right\}},$$

where

(20) $$a=\int_{0}^{0.6}\frac{5}{3}\;dx+\int_{0.6}^{255}\frac{1}{x}\;dx=1+\ln425,$$

and the one-sided Laplace distribution:

(21) $$f_{2}\left(x\right)=\ln\left(1.25\right)\;\cdot \;0.8^{x}.$$

**Figure 3:**
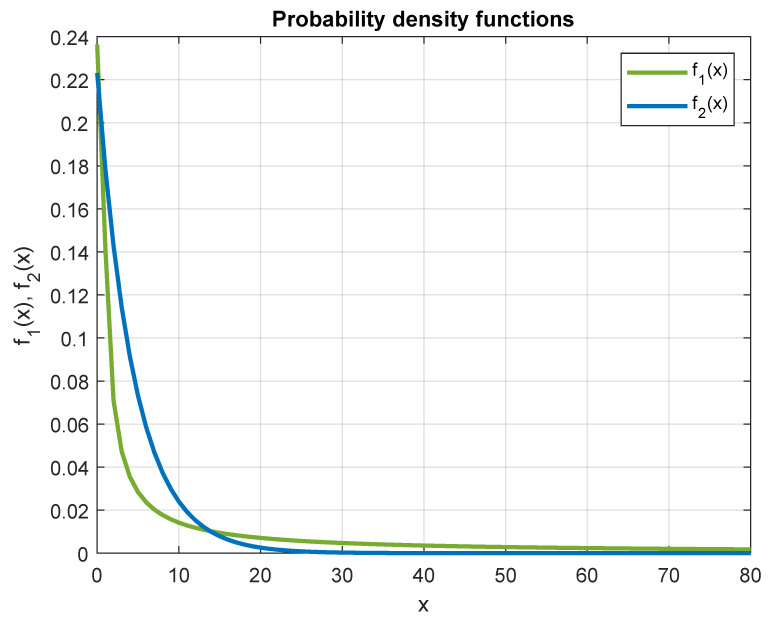
Comparison of $f_{1}\left(x\right)$ and $f_{2}\left(x\right)$ probability distributions.

The introduction of a new weight in the Formula (18) is, therefore, justified by the similarity of the shape of the graphs reflecting the probability distributions of $f_{1}\left(x\right)$ and $f_{2}\left(x\right)$ (as shown in Figure 3).

A second idea to improve the minimization of the bit average $L_{avg}$ using *IRLS* is to slightly reduce the weights $w_{n}$ for encoded pixels whose nearest neighborhood (or more precisely, elements of the $Y_{n}$ vector) are characterized by high variability (e.g., due to noisy data). We can calculate the level of this variability using the mean absolute deviation:

(22) $$D_{n}=\frac{1}{r}\sum_{i=1}^{r}\left|y_{n,j}-{\bar{y}}_{n}\right|,$$

where ${\bar{y}}_{n}$ is the arithmetic mean of the elements of vector $Y_{n}$. Then, Formula (18) is extended to the form:

(23) $${∥e∥}_{1}=\sum_{n\in Q}\frac{0.8^{e_{n}^{old}}}{\frac{200}{MAE_{old}}+D_{n}}\;\cdot \;{\left|e_{n}\right|}^{2},$$

where *MAE*_*old*_ denotes the mean absolute error obtained in the previous *IRLS* iteration (in the first iteration, it is initialized with $MAE_{old}=10$). In summary, the corrections made here in the form of a new weight:

(24) $$w_{n}={\left(\frac{0.8^{e_{n}^{old}}}{\frac{200}{MAE_{old}}+D_{n}}\right)}^{\frac{1}{2}},$$

we can refer to as *IRLS*_+_.

#### 2.4.3. Effectiveness of Improvements

By using *IRLS*_+_ and improving the *GBSW*_+_ method with the help of Formula (7), we were able to improve the bit average and encoding/decoding time relative to the *ALCM*_+_ codec presented in our previous work [27], which used the same Formula (3) for the predicted value and the same number of five iterations of scanning (pre-coding) the entire image to determine the prediction coefficients. The decoding time (Dell Precision 5690 mobile workstation (Dell, Round Rock, TX, USA) with Intel^®^ Core^™^ Ultra 7 165H processor, 3.8 GHz) of a Lennagrey image with a resolution of 512×512 pixels decreased by about 12% to 0.133 s for the order of r=14 and to 0.152 s at r=24. It should be noted here that the decoding time is linearly dependent on both the number of image pixels and the prediction order. On the other hand, the coding time decreased by 29.7% (0.671s) at r=14 and by 16.9% (1.01s) at r=24. Due to the non-linear dependence of coding time on the prediction order, a more detailed analysis of this aspect will be presented in Section 3.2.

Table 1 compares the bit averages obtained by the proposed *IRLS*_+_ method with other methods of determining prediction coefficients (using the same prediction error encoder). Two sets of most popular standard test images were used for testing. The first consists of 9 images and the second consists of 45 images; they are a subset of the entire image database available at [33].

## 3. Context-Dependent Linear Prediction

Section 2.4 presents how to determine the prediction coefficients that form the vector $B=[b_{1},b_{2},...,b_{r}]$ to be passed as header information to the decoder. Assuming that the prediction coefficient is between −1.999 and 1.999, we can write each coefficient on a certain small number of (N+2) bits (one bit each for the sign of the number and the integer value, respectively, and *N* bits of precision for the fractional part). In addition, since the sum of the coefficients is 1, it is sufficient to write only r−1 coefficients. For example, for an image with a resolution of 512×512 pixels at r=24, N=9, the header cost is only 0.000965 bits per pixel.

This is a small overhead, hence the idea to increase compression efficiency by expanding the prediction order and introducing a context-dependent predictor switching mechanism. Due to the variability of the neighborhood characteristics of the nearest pixel, it is possible to select several predictors individually tailored to the different types of neighborhoods of the currently encoded pixel. In the paper [34], we demonstrated the validity of this approach using simple context partitioning algorithms based on *MED* (Median Edge Detector—known from *JPEG-LS* [10]) and *GAP* (Gradient Adjust Predictor used in the *CALIC* codec [11]). In the first case, three and seven different contexts were obtained, and their corresponding predictors were obtained in the second case. Unlike the original versions of *JPEG-LS* and *CALIC*, where the predictors were fixed, in the work of [34], we used predictors selected individually for each image using the *MMSE* method. Although with seven contexts at N=9 and a higher prediction order r=80, the header cost for an image with a resolution of 512×512 pixels of 0.0232 bits per pixel is already noticeably higher, it is still acceptable. These are the upper limits on the number of contexts and prediction order we decided to introduce in the codec proposed in this work.

### 3.1. Algorithm to Determine the Context

In the case of using *GAP*, the main problem was the inefficient method of dividing seven contexts using a scalar quantizer with six fixed thresholds, which needed to sufficiently provide an individual for each image to match the dynamics of the digital signal.

In this work, we want to present a much more efficient contextual partitioning and use the *IRLS*_+_ algorithm discussed in Section 2.4 to determine the prediction coefficients for each context. Depending on user demand, the two-stage decision-making algorithm divides it into three and seven contexts.

In the case of splitting into three contexts to take into account the average level of variation of the digital signal, the average value from all the weighted variances *m* of the pixels closest to each encoded image pixel should be determined for a given image (Figure 1). We calculate the weighted variance from the formula:

(25) $${\tilde{\sigma }}^{2}=\frac{1}{\delta }\sum_{j=1}^{m}{\bar{d}}_{j}\;\cdot \;{\left(P\left(j\right)-\bar{p}\right)}^{2},$$

where the weights ${\bar{d}}_{j}$ are determined as the inverse of the Euclidean distance between pixels P(j) and P(0):

(26) $${\bar{d}}_{j}=\frac{1}{\sqrt{{\left(\Delta x_{j}\right)}^{2}+{\left(\Delta y_{j}\right)}^{2}}},$$

where $\Delta x_{j}$ and $\Delta y_{j}$ are the horizontal and vertical distances of pixel P(j) relative to P(0), respectively.

Furthermore:

(27) $$\delta =\sum_{j=1}^{m}{\bar{d}}_{j},$$

is the normalizing factor, while $\bar{p}$ is the arithmetic mean of the nearest neighbor:

(28) $$\bar{p}=\frac{1}{m}\sum_{j=1}^{m}P(j).$$

The first step in context partitioning is to quantize the surrounding weighted variance values ${\tilde{\sigma }}^{2}$ into three intervals (low, medium, and large variances) with thresholds of $\beta_{1}\;\cdot \;{\bar{\sigma }}^{2}$ and $\beta_{2}\;\cdot \;{\bar{\sigma }}^{2}$ (where ${\bar{\sigma }}^{2}$ is the arithmetic mean of all ${\tilde{\sigma }}^{2}$ weighted variances determined for the encoded image). Good results were obtained with m=10, $\beta_{1}=0.15$ and $\beta_{2}=1.2$. Determining the context number (denoted as ctx) from 0 to 2 is presented in detail by Algorithm 3.
**Algorithm 3** The first method of the context number calculation for pixel $x_{n}$ being encoded.1:Calculate the weighted variance (25):2:**if** $\left({\tilde{\sigma }}^{2}<\beta_{1}\;\cdot \;{\bar{\sigma }}^{2}\right)$ **then**3:    ctx←0;4:**else if** $\left({\tilde{\sigma }}^{2}<\beta_{2}\;\cdot \;{\bar{\sigma }}^{2}\right)$ **then**5:    ctx←1;6:**else**7:    ctx←2;8:**end if**

The second step is used when to obtain a split into seven instead of three contexts. For this purpose (in the situation of obtaining a mean or large variance), we check for a large discrepancy in the nearest neighborhood between the horizontal gradient $d_{h}$ and the vertical gradient $d_{v}$, where:

(29) $$\begin{array}{cc} d_{h}= & 2\;\cdot \;(\left|P(1)-P(5)\right|+\left|P(2)-P(3)\right|+\left|P(3)-P(7)\right|+\\ \; & \left|P(2)-P(4)\right|)+\left|P(6)-P(8)\right|+\left|P(6)-P(9)\right|\\ d_{v}= & 2\;\cdot \;(\left|P(2)-P(6)\right|+\left|P(1)-P(3)\right|+\left|P(4)-P(9)\right|+\\ \; & \left|P(3)-P(8)\right|)+\left|P(5)-P(7)\right|+\left|P(7)-P(11)\right| \end{array}.$$

The range of proportions $d_{h}:d_{v}$ allows us to divide the context (with medium or large variance) into three sub-contexts. In detail, the determination of the context number (denoted as ctx) from 0 to 6 is presented by Algorithm 4, where the additional parameter is set to $\beta_{3}=1.4$.
**Algorithm 4** The second method of the context number calculation for pixel $x_{n}$ being encoded.1:Calculate the weighted variance (25):2:**if** $\left({\tilde{\sigma }}^{2}<\beta_{1}\;\cdot \;{\bar{\sigma }}^{2}\right)$ **then**3:    ctx←0;4:**else**5:    Calculate gradients using (29);6:    **if** ($d_{h}>\beta_{3}\;\cdot \;d_{v})$ **then**7:        ctx←1;8:    **else if** $\left(d_{v}>\beta_{3}\;\cdot \;d_{h}\right)$ **then**9:        ctx←2;10:    **else**11:        ctx←012:    **end if**13:    **if** $\left({\tilde{\sigma }}^{2}<\beta_{2}\;\cdot \;{\bar{\sigma }}^{2}\right)$ **then**14:        ctx←ctx+1;15:    **else**16:        ctx←ctx+4;17:    **end if**18:**end if**

Depending on the number of contexts in the solution proposed here, perform Algorithm 3 or Algorithm 4 as an additional step in Algorithm 1 (between steps 1 and 2) and Algorithm 2 (between steps 3 and 4).

### 3.2. Selection of the Parameters of the Compression

#### 3.2.1. Impact of Prediction Order on Coding Time

The introduction of context splitting does not significantly affect the decoding time, which (for a Lennagrey image with a resolution of 512×512 pixels) in the seven-context version at r=10 is 0.145 s and 0.165 s at r=80.

**Figure 4:**
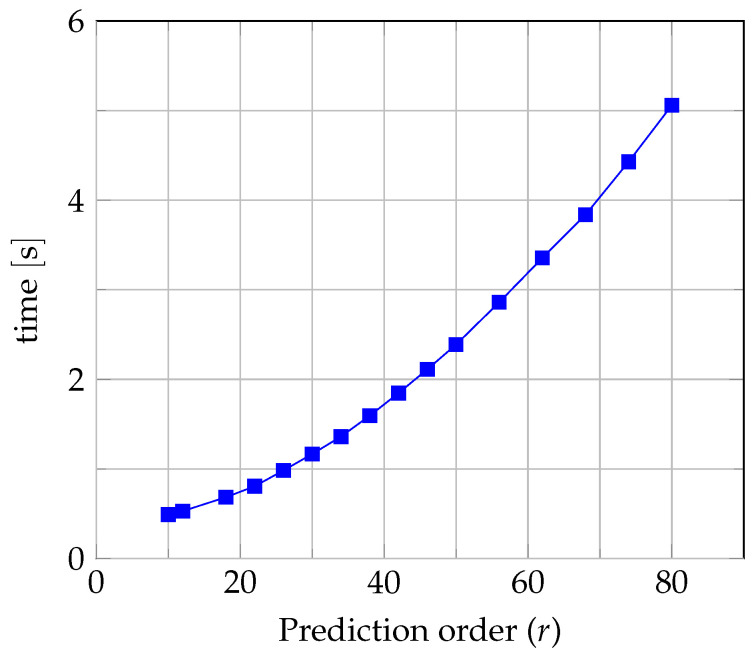
The encoding time as a function of the prediction order for a Lennagrey image with a resolution of 512×512 pixels.

The same is true for coding time, but there is a non-linear relationship concerning the prediction order. For the orders r=10 and r=80, the encoding times are 0.49 s and 5.06 s, respectively. Figure 4 presents an encoding time as a function of the prediction order.

Although as many as seven matrix equations must be solved in the seven-context case (see the Formula (12)), the time to solve them is negligibly short (24 μs at r=10 and 2559 μs at r=80) compared to the time to pre-determine the set of seven matrices R and vectors P. In the case of r=10, it is only 0.0285 s, but already at r=80 the time increases to 0.927 s, which, in this case, is more than 90 percent of the total image encoding time (it should be noted here that the procedure for calculating prediction coefficients in the $IRLS_{+}$ algorithm requires as many as five iterations). Since the R matrix is triangular, it is sufficient to fill its r(r+1)/2 elements. After taking into account the filling of *r* elements of the P vector, this requires a total of S(r(r+1)/2+2) multiplications and additions, where *S* is the number of pixels of the image (S>>r). For example, for an image with a resolution of 512×512 pixels at r=80 filling the R matrix and P vector requires $2^{9}\;\cdot \;2^{9}\;\cdot \;\left(80\;\cdot \;81/2+2\right)=849,870,848$ multiplications and additions. This work set the maximum order as r=80 as a compromise value, offering high efficiency with moderate coding time. It is important to emphasize that the encoding of an image is most often performed once by the user, and the decoding process is performed multiple times; hence, with r=80 from the user’s point of view, we obtain a good ratio of encoding versus decoding time of 30.77:1. Decoding time is linearly dependent on the prediction order and the number of pixels in the image.

#### 3.2.2. Cost of Prediction Coefficient Storage

Minimizing the bit average is a manageable problem due to the large number of variables affecting the final length of the encoded file. As the prediction order and the number of contexts increases, the mean absolute error can be expected to decrease. On the other hand, due to the requirement to save prediction coefficients, the size of the file header is negatively affected.

The solution proposed here defines three image classes in terms of size. Small, medium, and large images are characterized by their sizes: up to $2^{16}$ pixels, up to $2^{18}$ pixels, and counting above $2^{18}$ pixels, respectively. By using the Algorithms 1 and 2, we can adjust the size of the header for each of the three image classes by changing the number of contexts and the accuracy of the prediction coefficients, where each is saved using (N+2) bits. Table 2 presents the parameters for the images at three resolutions (each representing a separate class), with r=80.

For the smallest images of resolution 256×256, despite the significant reduction in the precision of the prediction coefficients to N=7 bits of the fractional part and the restriction to splitting into just three contexts, the header cost is not marginal, signaling the need for possible header data compression.

Although a reasonably wide range has been defined (from −1.999 to 1.999) for each of the coefficients, they are usually small values for which the arithmetic mean (after analyzing the probability distribution of a given coefficient) oscillates near zero. The higher the *j*th coefficient number $b_{j}$, the smaller its absolute value usually becomes. This can be used to compress coefficients using a context-dependent binary arithmetic encoder.

From a base of training images, approximate probability distributions can be constructed for each of the *r* coefficients. We then convert these distributions into probability distributions of the individual bits of a given coefficient (with required bit space of (N+2) bits). The sign bit of the coefficient is encoded first, followed by the bit specifying the integer part of the number (with a value of 0 or 1). Next, we encode *N* bits of the fractional part of a given coefficient sequentially from highest to lowest, respectively.

For the fractional part of the coefficient, we can introduce a contextual division that considers several distributions for a given bit by obtaining preliminary information about the previously encoded bits (used further as contextual bits). We first use the information in the two encoded bits to do this. The first is the sign bit ($s_{1}$) of the encoded coefficient. The second context bit is determined on the fly based on information about whether at least one bit with a value of 1 has ascended among the previously encoded most significant bits of a given coefficient, in which case $s_{2}=1$, otherwise $s_{2}=0$. This gives us a two-bit number $s_{1}s_{2}$ with values between 0 and 3, which, when multiplied by four times the bit number of the fractional part in the range from 1 to *N*, gives us the number of the context in which the binary distribution is stored (in practice, a single number storing the probability of occurrence of a bit with a value of 0 is sufficient) corresponding to the currently encoded bit. The encoder and decoder have a built-in database with a set of such $4\dot{N}$ distributions for each of the (r−1) prediction coefficients (with the first coefficient not encoded, since on the decoder side, it can be calculated by assuming that the sum of the coefficients of the B vector is 1).

Two facts are noticeable: First, the less significant the coefficient, the more random it is, i.e., it is less susceptible to compression. Second, the higher we set the prediction order, the lower the average cost per coded coefficient. Table 3 presents (obtained for the test image database) the average cost of saving one prediction coefficient depending on the precision of writing these coefficients using two cases for r=42 and r=80, respectively. It shows that with r=80, N=8, it was possible to reduce the header’s size by an average of 46.64%.

#### 3.2.3. Influence of the Choice of the Parameters on the Efficiency of Compression

In the proposed codec, using Algorithms 1 and 2, further supported by contextual partitioning (see Algorithms 3 and 4), referred to hereafter as the base version, several parameters were fixed. This is the prediction order r=80, the neighborhood size m=10 (see Formula (25)), three quantization thresholds: $\beta_{1}=0.15$, $\beta_{2}=1.2$ and $\beta_{3}=1.4$, and the five iterations in the *IRLS*_+_ algorithm. In addition, depending on the size of the images (small, medium, and large images of up to $2^{16}$ pixels, up to $2^{18}$ pixels and those counting more than $2^{18}$ pixels, respectively), two fixed parameters were set: the number of contexts (which is three for small images and seven in other cases) and the precision of the prediction coefficients save of 9 (for small), 10 (for medium) and 11 bits (for large images), respectively.

In addition to the proposal for encoding of the prediction coefficients (which the base version of the encoder does not use), discussed in Section 3.2.2, it is possible to reduce the bit average by individually adjusting the eight encoder parameters listed here to the characteristics of a particular image. However, this requires multiple image encodings. Additional ideas for improvement can be introduced, requiring additional iterations of image encoding (such as choosing the correct parameters in the arithmetic encoder). To ensure that the entire tuning stage takes little time and that the modified parameter information itself does not unduly increase the size of the header, it is necessary to narrow the search area and determine their respective order of parameter selection. For this purpose, we designed an Algorithm 5 to select the best compression parameters.

Disregarding the last (12th) step of the Algorithm 5, more than 2 million combinations of parameter settings listed in steps 2 through 11 are possible. However, executing the individual steps of the Algorithm 5 appropriately limits the number of tests (involving full image encoding) to just 43.
**Algorithm 5** Tuning compression parameters to reduce the bit average.1:Set start parameters according to base version (except for one less number of iterations within the $IRLS_{+}$ algorithm).2:Calculate the one of best of 8 lossless phases of image rotation (at r=22).3:Calculate the best class of prediction order (three tests, one representative each from the following classes, respectively: r=18 for the low-order class, r=42 for the medium-order class, and r=68 for the high-order class) for the version with seven contexts.4:Repeat step #3 for the three-context version, choosing a winner within steps #3 and #4.5:Perform more accurate prediction order selection (five tests) for the winning class.6:Choose the best parameter *m* (see Formula (25)) from the set $\left\{8,10,12,28\right\}$.7:Choose the best pair of quantization thresholds $\left\{\beta_{1},\beta_{2}\right\}$ from a set of seven settings.8:Choose the best accuracy for saving prediction coefficients on *N* bits after the decimal point from the set $\left\{6,7,8,9\right\}$.9:If the seven-context version is chosen, select the best quantization threshold $\beta_{3}$ from the set $\left\{1.3,1.4,1.5,2.4\right\}$10:Check if the version with the CDCCR block disabled (with $C_{mix}=0$) improves the bit average.11:Check how many iterations at the $IRLS_{+}$ stage gives the best result (nine tests).12:Perform a set of arithmetic coding tests for different encoder settings.

Due to the slight difference in efficiency between four and five iterations in *IRLS*_+_, it was decided to reduce (relative to the base version of the encoder) to four iterations as a first step, which reduces the time of most tests by almost 20%.

Each rectangular image can be rotated losslessly in $90^{\circ }$ increments, resulting in four different settings. Suppose we combine this with the mirroring capability. In that case, we obtain a doubling of these lossless image transformations (which the decoder can perform in reverse, restoring the original image). This transformation can affect the final compression efficiency due to the different neighborhood selections and encoded pixel order. Therefore, eight turnover phase selection tests are performed in step 2 (with a relatively small but representative order r=22).

Steps 3 and 4 are where the decision is made to choose the number of contexts: three or seven. To reduce the total number of tests for selecting a prediction order (from the considered 16-element set of orders between 10 and 80), an initial selection of this order is made from among the three possibilities of $\left\{18,42,68\right\}$ and twice, as both the three-context and seven-context versions. If the bit average is lowest for r=18, in step 5, we make a complementary fine-tuning of the order from the set $\left\{10,12,22,26,30\right\}$. On the other hand, when the order r=42 or r=68 won in steps 3 and 4, the sets of complementary orders in step 5 are, respectively: $\left\{30,34,38,46,50\right\}$ and $\left\{50,56,62,74,80\right\}$. As a result, instead of 32 tests (16 different orders each in the 3-context and 7-context versions), only 11 tests are performed in steps 3–5.

In step 6, the best parameter *m* is selected from a set of $\left\{8,10,12,28\right\}$, and in step 7, the best pair of quantization thresholds $\left\{\beta_{1},\beta_{2}\right\}$ is selected from a set of 7 experimentally selected settings: $\left\{\left\{0.02,1.00\right\},\left\{0.08,0.80\right\},\left\{0.08,1.00\right\},\left\{0.10,1.00\right\},\left\{0.10,1.20\right\},\left\{0.15,1.20\right\},\left\{0.30,1.40\right\}\right\}$. Step 8 allows us to choose the best accuracy for saving the prediction coefficients at *N* decimal bits from the set of $\left\{6,7,8,9\right\}$, taking into account the compression of the prediction coefficients discussed in Section 3.2.2. Step 9 is performed if the decision to use the division into seven contexts was previously made. The quantization threshold $\beta_{3}$ is tuned from the allowed $\left\{1.3,1.4,1.5,2.4\right\}$. Step 10 is just one test to see if the version with the CDCCR block disabled (i.e., with $C_{mix}=0$) will improve the bit average, which happens less than 10% of the time.

The final (11) step affecting the determination of the final set of prediction coefficients is the selection of the number of iterations in *IRLS*_+_, where we check it in the range of 3 to 10. After the first iteration, the result is also considered, corresponding to the classic *MMSE* method.

Step 12 involves only minor modifications to the parameters of the arithmetic encoder. Although there are quite a lot of tests, 140, they are performed relatively quickly, as they use the already coded prediction error map created after step 11 (unlike the earlier steps of the Algorithm 5, the time-consuming step of determining a new set of prediction coefficients based on *IRLS*_+_ is absent here). Information about the winning parameters is just a few extra header bytes.

Table 4 shows the impact of disabling individual parameter tuning steps within the Algorithm 5. The results show the bit average’s growth level based on averaged results for 45 test images. It should be emphasized that the actions in the individual steps to be turned off affect each other, so these losses should not be directly summed up to assess the overall suitability of the Algorithm 5. Using Algorithm 5 allowed us to improve the bit average by 0.02679 bit/pixel on average.

A check was also made on the usefulness of introducing the denominator weight $w_{n}$ in the Formula (24). The gain averaged 0.001 bit/pixel.

To estimate the implementation complexity of the process of encoding a Lennagrey image with a resolution of 512×512 preceded by the execution of the selection of the best parameters (all steps of the Algorithm 5), we introduced forced actions to be able to analyze two extreme situations. In the first (fastest) case after step 2 of the Algorithm 5, we forced the transfer of the winning order r=18, and after step 4, the triple-context version with order r=10 was indicated as the winner. The coding time was 58.1 s. In the second (slowest) case after step 2 of the Algorithm 5, we forced the transfer of the winning order r=68, and after step 4, the 7-context version with order r=80 was indicated as the winner. In this case, the encoding time has increased to 184.7 s.

## 4. Experiments and Analysis of the Effectiveness of Existing Solutions

This section compares the encoding and decoding time and efficiency of the proposed method and other well-known classical codecs (without the machine learning category) as representing the deep learning category.

### 4.1. Analysis of Encoding and Decoding Time

Today’s computer systems, with the development of technology, are becoming less and less stable every year regarding reliable measurement of coding time. This is mainly due to multicore (which only a few codecs take advantage of, as most perform their tasks sequentially concerning single image compression). But even more problematic are the mechanisms for auto-adjusting the CPU clock frequency depending on the number of currently running computing threads and the current CPU temperature (which depends on the performance of the automatically regulated cooling system).

For example, an attempt to use a computer Dream Machines (Warsaw, Poland) NP50DE (Windows 10, 16 GB RAM) with a six-core Intel i7 10750H processor with a nominal frequency of 2.6 GHz (Windows 10, 16 GB RAM) for encoding time measurements showed that running a single encoder causes the clock frequency to oscillate around 4.7–4.8 GHz (thanks to Turbo mode, the nominal value was significantly exceeded), decreasing during moments of increased data transfer from the SSD to 3.7GHz (during the final stage under Algorithm 5 involving multiple encoders tuning parameters in an adaptive arithmetic encoder).

To increase the measurement stability of the encoding/decoding time, we used an up-to-date Dell Precision 5690 mobile workstation (Intel^®^ Core^™^ Ultra 7 165H processor, 3.8 GHz, 32 GB DDR5, 2 TB SSD, Windows 11), powered by an external power supply, with the ultra performance option set in Dell Optimizer management application. In addition, only one user application was run (minimizing the level of disturbance to the stability of the total load on the quad-core processor by any applications executing in the background). The time measurement was calculated as the arithmetic average of a sequentially repeated 100 or 1000 times encoding/decoding process.

In contrast, the difficulty of practically assessing encoding times based on theoretical, computational complexity nowadays stems from the use of highly efficient processor caches (the impact of which on the variation of measured time can be counted in the hundreds of percent depending on the arrangement of memory references in a given algorithm), as well as the possible opportunities of parallelizing encoding in the era of commonly used multicore processors. Despite these caveats, we have presented an analysis of both encoding and decoding times and the number of multiplications and additions for the most complex operation of processing encoded data in Section 3.2.

In recent publications, the issue of measurement reliability can be further complicated by the possibility of using highly specialized, multi-core graphics cards, which already happens with some of the latest solutions using neural networks. This aspect will be discussed more extensively in the next section.

### 4.2. Overview of Deep Learning-Based Solutions

Since a whole branch of lossless image compression methods based on deep learning using neural networks has been developed in recent years, we have decided to devote a separate section to this issue, where we want to highlight some of the drawbacks of this type of solution.

The first works using neural networks for lossless image encoding used an adaptive method in which the network weights are modified on the fly after each successive pixel is encoded (backward-adaptive methods)—this is learning the network on the fly without pre-learning using training image bases. In the work of [12,13,14], an Adaptive Neural Network (AdNN) was used, whose design was based on the Multilayer Perceptron Network (MLP), while in the work of [15], Cellular Neural Networks (CNNs) were used. Our attempts to achieve the highest possible compression efficiency (without pre-learning the network) using AdNN enhanced with context-sensitive switching showed that using such networks in a backward-adaptive version requires high implementation complexity of the encoder and decoder, comparable to that of *WLS*, with the bit-average results obtained with neural networks proving to be noticeably inferior to *WLS* [35].

Other, newer approaches based on neural network deep learning are widely described in papers [36,37]. Different categories exist within the entire pool of state-of-the-art codecs using deep learning with neural networks. For example, the work of [38] proposes a combination of lossy *BPG* coding (which generates a map of ${\hat{x}}_{n}$ prediction values) and lossless prediction error compression (an idea known even from the work of [8]). In contrast, the work of [39] uses sub-image (lower resolution) partitioning, where one of the sub-images is encoded using classic *JPEG-XL* (this approach is known from the original version of *CALIC*, and also used in multi-resolution codecs using wavelet transforms), and only the remaining data are encoded using deep neural networks (*LC-FDNet*).

Unfortunately, in the case of solutions based on neural networks, the authors usually skip the analysis of the entire literature (without noticing the duplication of ideas, for example) on lossless image compression – calling Non-Learned Methods as classical approaches or codecs (for short) without learning. Just as often, they seem to overlook the distinction between encoding and decoding times (e.g., the paper [37] presented a graphical comparison of only the summed encoding and decoding times for 11 codecs). In doing so, using an interpretable programming language such as Python 3, in many cases, does not facilitate the reliable analysis of encoding/decoding times against fully compiled and optimized classical codecs (e.g., *CALIC*, *WebP*). Even in the paper [40] summarizing the achievements of deep neural networks, there needs to be a comparative mention of the encoding and decoding times of the solutions discussed there.

In the work of [41], we can come across a comparison of several neural network-based solutions, including the conclusion that *PixelCNN*-type methods (e.g., [42]) are generally computationally expensive. In addition, a disadvantage of these solutions is the strong dependence on the learning base of such networks (it is easy to have a network overfitting effect, especially for images with very low resolution 32×32 or 64×64, resulting in too low efficiency in more comprehensive application compared to classical solutions), which was criticized in the paper [43].

Based on bibliography data, published proportions of coding times can be used. For the work of *IDF*, [44] shows that the *IDF* [41] method is (depending on the image base) 63 to 143 times slower concerning *WebP* encoding (for decoding, the ratio is even more unfavorable, ranging from 4857:1 to as much as 89,043:1). Against this background, the GPU-processing-based *L3C* [38] codec (1.5 to 3.33 times slower than *WebP* at the encoding stage and between 110 and 1614 at the decoding stage) and *SReC* (2.1 to 2.5 times slower than *WebP* at the encoding stage and between 338 and 1643 at the decoding stage) fare significantly better. In contrast, measurements presented by the authors of *L3C* [38] (most likely due to the use of a suitably robust GPU system) show a 1.5:1 encoding time ratio and a 5.25:1 decoding time ratio relative to *WebP* (for a 512×512 pixel image). At the same time, the work of [45] showed that for a set of classic Kodak test images (24 images with a resolution of 768×512 pixels), the *L3C* codec proved not only slower but achieved lower lossless compression efficiency compared to *WebP 1.0.2*. Much, therefore, depends on the selected set of test images, and in the modern world, it is rare to find pictures with such small resolutions as 32×32 or 64×64 preferred by the “deep learning world”. Confirmation of this uncomfortable conclusion can be found in the work of [46], where explicitly, the authors state that for high-resolution images, conventional codecs still have an advantage (offering a lower bit average) over learning-based methods. Another research team in the paper [36], after an in-depth analysis of the deep neural networks (DNNs) literature, also came to the opinion that existing lossless image methods based on learning tend to suffer from too low coding speed and are difficult to apply to practical image compression tasks at full resolution.

In a paper by [47], the authors boasted the higher efficiency of their *HiLLoC* solution relative to *IDF* while showing the possibility of parallelizing the coding process. The only published result of 500×374 image encoding time is about 29 s (on a six-core computer with GTX 1060 GPU support).

The paper [36] also did not specify the CPU clock, instead comparing several codecs for moderately high-resolution images (against other papers on the subject), including 768×512 pixels, where the authors presented a method *DLPR* characterized by high efficiency (for moderately high-resolution images) and 6.5 times shorter encoding time of 1.26 s (and 4.4 times shorter decoding time of 1.8 s) relative to *L3C*. Interestingly, the results of testing the same L3C codec can be found in the work of [48], where the encoding time (for images with a resolution of 512×512 pixels) was found to be 12.8 times shorter than for *WebP 1.0*. Again, the authors should have specified which hardware the experiments were conducted on. However, this shows a discrepancy in the conclusions about encoding/decoding times that can occur depending on the GPU technology used, which the *L3C* codec uses.

In the work [49], we obtained reliable information about the architecture of the hardware used for testing: NVIDIA GeForce RTX 2080 GPU + Intel i7-9700 CPU (8 cores, 3.0/4.7 GHz in Turbo mode). From this research, the average encoding/decoding times of an image with a resolution of 512×512 pixels using *L3C* are, respectively, 0.49/0.54 (unfortunately, no comparison with the encoding times of classical solutions is included). In the test mentioned above, the authors also present their own *LPPLIC* solution, where they honestly demonstrate how much, in the case of methods using neural networks, the encoding and decoding time depends on the ability to parallelize the algorithm and the use of GPU technology to support this parallelization. In the sequential implementation of the decoding algorithm, the ratio of decoding time to encoding time is 433.5:1 (with the decoding time for an image with a resolution of 512×512 pixels being 711.03 s, with the *LPPLIC* decoding time decreasing to 26.26 s when using the parallelized version).

None of those mentioned above indicate which compression option was used for *WebP* (representing the classic codecs in the comparisons), directly affecting its compression efficiency (bit average) and coding time. Only the authors of the paper [50] indicated that they used the switch offering the shortest encoding time for compression (which translates into less than the minimum bit average that *WebP 1.2.1* can offer). They also changed the original encoder to their own to speed up encoding in *L3C*.

In summary, the above analysis of recent work indicates that the ability to parallelize the encoding/decoding process and GPU performance plays a crucial role in methods with learning (using neural networks). Versions of eminently sequential algorithms require (compared to classical solutions) very long encoding and especially decoding times. Despite the shortcomings mentioned here, the next section will analyze several representatives of DNN-type solutions in terms of compression efficiency against classical solutions (including our proposed solution).

### 4.3. Comparison of the Effectiveness of the Proposed Solution

In Section 3, we proposed a new way to compress images based on, among other things, context splitting and *IRLS*_+_ minimization. Because it is time asymmetric (with a reasonably short decoding time), we proposed two versions of the encoding: a baseline (with a fixed set of parameters, such as the prediction order r=80) and an extended version that includes a pre-selection of parameters (12 steps of the Algorithm 5). An analysis of the encoding time shows that for an image with a resolution of 512×512 pixels, the encoding time of the base version is 5.06 s, and in the enhanced version it varies within 36.6–116.36 s. Concerning encoding/decoding time measurements, it should be noted that the current C-language codec implementation has not been parallelized, the code optimization process has not been performed, and the latest technologies, such as those using the GPU, have not been used. At the same time, it should be noted that due to its simplicity, the decoding algorithm lends itself to easy hardware implementation.

The advantage of the proposed solution is the short decoding time, which is (depending on the prediction order) between 0.145 s and 0.165 s for the Lennagrey image, which can be considered an acceptable value. Although faster solutions offer faster encoding and decoding times (e.g., *JPEG-LS* or *WebP*), they cannot compare in terms of compression efficiency. Table 5 compares the bit averages for nine most popular test images of the proposed solution with those obtained by several methods known from the literature with fast encoding and decoding times. Based on this comparison, it can be seen that our proposed solution offers, on average, 9.1% lower bit average against *JPEG-LS* and 8.7% against *WebP 2* (in the highest efficiency mode using the following settings: “-q 100 -alpha_q 100 -effort 9”.

On the other hand, among time-symmetric solutions with a comparable, albeit slightly higher, bit average (relative to our solution), one can point out, for example, such as *Glicbawls* [51], where the decoding time is 4.47 s). Among the methods using neural networks, there is an extensive range of encoding times; one example characterized by a reasonable time can be *LCIC*_duplex_ (1.14 s on an i5-7600 processor 3.5 GHz) [37]. Again, the proposed solution offers, on average, a 4.55% shorter bit average, as presented in Table 6, where a comparison of both classical codecs (no-learning category) and those representing the deep learning category (*L3C*, *CWPLIC*, *LCIC*_duplex_) could be found.

## 5. Conclusions

Based on the assumption that we usually encode an image once and decode it multiple times, users of computer systems should care about a reasonably fast decoding process. Therefore, in this work, we proposed a time-asymmetric method that uses linear and nonlinear prediction with forward adaptation at the data modeling stage. This treatment yields prediction errors with a high susceptibility to compression using adaptive variations of the Golomb code and a binary arithmetic encoder.

The solution proposed here uses an improved *IRLS*_+_ method to determine linear prediction coefficients, which allows the mean absolute error to be minimized. In addition, high compression efficiency was achieved by using the proposed context-switching algorithm, which allows the use of several prediction models tailored to the individual characteristics of each image area. In addition to the base version (with a relatively low encoding time despite the lack of need for highly efficient GPU systems, as is often the case with the latest deep learning-based solutions), we also presented an optional version extended with a method of pre-selecting compression parameters to increase its efficiency (in both cases, a satisfactorily short decoding time was achieved). Our proposed solution offers, on average, 9.1% lower bit average against *JPEG-LS* and 8.7% against *WebP 2*.

## Figures and Tables

**Table 1:** Comparison of bit averages in bits per pixel (bpp) for two sets of standard test images and two prediction orders, obtained by proposed $IRLS_{+}$ method with other methods of prediction coefficient calculation.

Image	MMSE r=14	${\mathrm{ALCM}}_{+}$ [27] r=14	${\mathrm{IRLS}}_{+}$ r=14	MMSE r=24	${\mathrm{ALCM}}_{+}$ [27] r=24	${\mathrm{IRLS}}_{+}$ r=24
Balloon	2.742	2.748	2.731	2.733	2.733	2.720
Barb	4.238	4.238	4.210	4.221	4.211	4.196
Barb2	4.416	4.421	4.403	4.406	4.401	4.393
Board	3.446	3.449	3.426	3.436	3.425	3.417
Boats	3.717	3.711	3.694	3.707	3.696	3.688
Girl	3.612	3.624	3.594	3.609	3.612	3.595
Gold	4.310	4.304	4.298	4.299	4.293	4.290
Hotel	4.180	4.176	4.151	4.179	4.169	4.153
Zelda	3.634	3.620	3.621	3.621	3.598	3.603
Avg. (9 images)	3.810	3.810	3.792	3.801	3.793	3.776
Avg. (45 images)	4.045	4.038	4.025	4.032	4.021	4.007

**Table 2:** Header cost depending on image size at order r=80.

Resolution	256×256	512×512	720×576
Number of contexts	3	7	7
(N+2)	9	10	11
Header cost [b/pixel]	0.03255	0.02110	0.01467

**Table 3:** Average bit numbers of the prediction coefficient after arithmetic coding as a function of input data word length.

Coefficient Precision (N+2) Bits	8	9	10	11
Average cost (r=42)	3.96113	4.90561	5.88912	6.88633
Average cost (r=80)	3.46996	4.37203	5.33586	6.32542

**Table 4:** Influence of disabling individual steps of Algorithm 5 on the average degradation of the bit average (for 45 test images).

Detachable Fine-Tuning Step in Algorithm 5	Step Number	Average Degradation in b/pixel
Rotating phase	2	0.00623
Order and number of contexts	3, 4, 5	0.00210
*m* parameter	6	0.00172
Quantization levels $\left\{\beta_{1},\beta_{2},\beta_{3}\right\}$	7, 9	0.00236
Precision of prediction coefficients	8	0.00032
Additional coding of prediction coefficients	8	0.00449
Testing of disabling CDCCR block	10	0.00315
Selection the iteration number of *IRLS*_+_ step	11	0.00268
Parameters selection of the arithmetic coder	12	0.00688

**Table 5:** Comparison of bit averages (for nine standard test images) obtained by known methods.

Image	*WebP 2*	*JPEG-LS*	*CALIC*	*JPEG* *XL*	*Blend-7* [29]	*HBB* [28]	*ALCM*_+_ [27] r=24	*Glicbawls* [51]	Prop. Soln (Base)	Prop. Soln (+Alg. 5)
Balloon	2.92	2.889	2.78	2.757	2.84	2.80	2.733	2.640	2.622	2.617
Barb	4.51	4.690	4.31	4.271	4.43	4.28	4.211	3.916	4.049	4.035
Barb2	4.67	4.684	4.46	4.483	4.57	4.48	4.401	4.318	4.303	4.292
Board	3.64	3.674	3.51	3.497	3.57	3.54	3.425	3.392	3.336	3.317
Boats	3.94	3.930	3.78	3.786	3.84	3.80	3.696	3.628	3.605	3.591
Girl	3.89	3.922	3.72	3.696	3.76	3.74	3.612	3.565	3.528	3.520
Gold	4.45	4.475	4.35	4.352	4.42	4.37	4.293	4.276	4.243	4.235
Hotel	4.37	4.378	4.18	4.193	4.29	4.27	4.169	4.177	4.084	4.067
Zelda	3.81	3.884	3.69	3.695	3.79	3.72	3.598	3.537	3.544	3.531
*Average*	4.022	4.058	3.864	3.859	3.946	3.889	3.793	3.717	3.702	3.689

**Table 6:** Measurement of bit average values—second set of test images [37].

Image	*BPG*	*PNG*	*LCIC*	*JPEG* *2000*	*JPEG* *LS*	*JPEG* *XL*	*FLIF*	*WebP 2*	*L3C*	*CWPLIC*	*LCIC* Dupl. [37]	Prop. Soln (+Alg. 5)
Airplane	4.32	4.26	3.99	4.00	3.80	3.71	3.82	3.84	4.56	3.69	3.69	3.563
Barbara	5.06	5.22	4.61	4.61	4.70	4.40	4.56	4.51	5.44	4.35	4.36	4.046
Coastguard	5.70	5.06	4.82	4.83	4.86	4.73	4.93	4.82	5.82	4.80	4.83	4.338
Comic	6.15	5.84	5.63	5.65	5.30	5.07	5.50	5.39	6.60	4.83	4.83	4.805
Flowers	5.18	5.08	4.91	4.92	4.62	4.51	4.74	4.70	5.53	4.41	4.35	4.338
Goldhill	4.95	4.70	4.58	4.59	4.43	4.37	4.50	4.41	5.27	4.33	4.33	4.175
Lennagrey	4.54	4.61	4.31	4.31	4.24	4.16	4.28	4.13	4.95	4.13	4.08	3.955
Mandrill	6.61	6.23	6.11	6.11	6.04	5.98	6.14	5.90	6.97	5.95	5.89	5.724
Monarch	4.10	4.26	3.82	3.82	3.70	3.54	3.68	3.72	4.37	3.40	3.45	3.359
Pepper	4.77	4.90	4.63	4.63	4.51	4.48	4.58	4.47	5.38	4.67	4.38	4.189
Ppt3	2.20	2.35	2.41	2.41	2.04	1.84	1.87	2.01	3.71	2.14	2.07	1.769
Zebra	5.83	5.19	4.89	4.89	4.81	4.66	4.84	4.84	6.08	4.65	4.68	4.363
*Average*	4.951	4.808	4.559	4.564	4.421	4.288	4.453	4.395	5.390	4.279	4.245	4.052

## Data Availability

The data sets for this study are available upon request from the corresponding author.
